# Compassion fatigue among frontline healthcare workers during the covid-19 pandemic in Tunisia

**DOI:** 10.1371/journal.pone.0276455

**Published:** 2022-10-27

**Authors:** Nihel Omri, Olfa Ezzi, Asma Ammar, Wafa Benzarti, Dorra Loghmari, Emna Toulgui, Asma Ben Abdelkarim, Asma Boukadida, Mansour Njah, Mohamed Mahjoub

**Affiliations:** 1 Department of Infection Control, Farhat Hached University Hospital, Sousse, Tunisia; 2 Faculty of Medicine of Sousse, Sousse, Tunisia; 3 Department of Pneumology, Farhat Hached University Hospital, Sousse, Tunisia; 4 Emergency Medical Services, Sahloul University Hospital, Sousse, Tunisia; 5 Department of Physical Medicine and Rehabilitation, Sahloul University Hospital, Sousse, Tunisia; 6 Emergency Department, Farhat Hached University Hospital, Sousse, Tunisia; 7 Department of Endocrinology Diabetology, Farhat Hached University Hospital, Sousse, Tunisia; KIMS: Kalinga Institute of Medical Sciences, INDIA

## Abstract

**Background:**

Healthcare workers (HCWs) are highly vulnerable to compassion fatigue (CF), which not only leads to decreased mental and physical health, but also to deterioration in the safety of care delivered. Our study aims to measure compassion satisfaction (CS), CF levels and their predictors among Tunisian HCWs.

**Methods:**

We conducted a cross-sectional study among HCWs caring for confirmed and suspected Covid-19 patients, staff at two university hospitals in Sousse, Tunisia during the 4^th^wave of coronavirus through a self-administrated Questionnaire, using the French version of the Professional Quality of Life scale ProQol, version 5.

**Results:**

A total of 274 professionals were recruited with a mean age of 32.87±8.35 years. HCWs tend to have an overall moderate levels of compassion satisfaction, secondary traumatic stress and burnout with mean scores 35.09±7.08, 29.72±7.62, 28.54±5.44 respectively. Self-reported resilience (β = 0.14, p = 10^−3^), work engagement (β = 0.39, p = 10^−3^) and burnout (β = -0.32, p = 10^−3^) were the predictors of compassion satisfaction in the linear regression analysis (adjusted r^2^ = 0.45). Similarly, limited work experience, compassion satisfaction and secondary traumatic sub-scores were the determinants of burnout (β = -0.1, p = 0.04; β = -0.54, p = 10^−3^; β = 0.35, p = 10^−3^ respectively); (adjusted r^2^ = 0.48). Regarding STS, female professionals (β = 0.20, p = 10^−3^), being married (β = 0.19, p = 10^−3^), the fear of transmitting the infection (β = 0.11, p = 0.03) and burnout (β = 0.39, p = 10^−3^) were the predictors for the occurrence of secondary traumatic stress (adjusted r^2^ = 0.48).

**Conclusion:**

More resilience promoting interventions and more coping skills programs must be implemented to fulfill HCWs’ psychological well-being needs.

## Introduction

The Covid-19 pandemic spread rapidly around the world beginning in December 2019, confining entire populations, filling overcrowded hospitals with mass arrivals of patients with severe forms of the disease and resulting in a dramatic increase in mortality [1]. It has turned into the most significant health crisis of the current era [2].

The emerging literature had identified the Covid-19 crisis as a public mental health issue [3]. In fact, it was regarded as an unpleasant experience causing numerous psychological distresses such as anxiety, depression, panic and post traumatic stress disorders among those who undergo it and especially among healthcare workers (HCWs) who were on the front lines against Covid-19 [4, 5].

Health institutions were being challenged by the pandemic: some HCWs were on the front lines especially those in departments caring for suspected or confirmed Covid-19 patients, others were dealing with the reorganization of the health system that such a pandemic requires [1, 6]. Indeed, physical exhaustion associated with reorganization of work spaces or adaptation to rigid work organizations, risk of contamination, limited resources, high patient mortality and particularly the feeling of loss of control following the lifting of the lockdown and the sharp increase in morbidity and mortality have led HCWs to experience high levels of secondary traumatic stress and burnout both of which are included in a broader term, compassion fatigue (CF) [7].

Hence, because of their overexposure to patient suffering and distresses affecting negatively their ability to be compassionates, HCWs may develop CF which was defined by Sabo et al. as “*a state of emotional exhaustion due to the dynamics of a caring relationship with an individual or a group of individuals who have suffered a sudden or severe loss*” [8]. Indeed, it corresponds to the decreased ability to empathize with patients due to overexposure to their suffering [9]. It usually occurs after a period of time in which the HCW has devoted a great deal of energy to caring for the patients in their care [10].

Moreover, CF being widely described as a syndrome closely related to traumatic situations, if left unmanaged, it can have unfortunate repercussions on HCWs, resulting in two components: burnout (BO) and secondary traumatic stress (STS) [11].

BO was defined by Maslach et al. as *“a state of psychological*, *emotional*, *and physical stress in response to prolonged exposure to occupational stress”* [12, 13]. Secondary traumatic stress refers to *“a condition in which trauma or stressful events is witnessed but not actually experienced”*. Thus, Stamm et al. integrated both of them into a broader concept, quality of work life (ProQoL), with compassion satisfaction (CS) which refers to the positive feelings about people’s ability to help others [7, 14]. In fact, professional quality of life is about emotions that an individual encounters in his/her job as a helper, it incorporates a combination of positive (compassion satisfaction) and negative aspects (secondary traumatic stress and burnout) [15, 16].

Analyzing the prevalence of CF, conceptualized in burnout and secondary traumatic stress leads us to explore a critical term “resilience” which is a dynamic process of positive coping despite experiencing traumatic events and exhaustion [16, 17]. In fact, it has been recognized as a key protective factor against the effects of CF among HCWs in numerous studies worldwide [18].

With the ongoing fight against Covid-19and the emergence of new variants, available researches have revealed that frontline HCWs will continue to experience high rates of CF, primarily those working in intensive care units, emergency departments and Covid-19 units [19, 20]. Thus, gaining a better understanding of the extent to which HCWs’ quality of work life is affected by CF during the Covid-19 outbreak seems to be critical for the development of a positive practice climate. In this context, the professional quality of life among Tunisian HCWs during the Covid19 pandemic remains poorly known. So, our study seems to be one of the first to address this subject. It aims to measure the level of positive (compassion satisfaction) and negative (secondary traumatic stress and burnout) aspects of professional quality of life ProQoL and to determinate predictor factors of each component among HCWs staff at two University Hospitals in the governorate of Sousse, Tunisia.

## Methods

### Study design and participants

We carried out a cross-sectional study among HCWs staff at the university Hospitals Farhat Hached and Sahloul, Sousse which is located in the Central-East part of Tunisia. The study was performed during the 4^th^wave of coronavirus in Tunisia (June, July and August 2021) using an anonymous and self administrated questionnaire. Only HCWs caring for confirmed or suspected Covid-19 patients were eligible to participate in this study.

### Measurement

#### Professional quality of life scale, PROQOL, version 5

Is a 30-items scale assessing positive and negative aspects of professional quality of life among HCWs [16]. Quality of work life is about emotions that an individual experiences in his or her work of helping others [14]. In fact, Consisting in three subscales: compassion satisfaction, secondary traumatic stress and burnout, PROQOL, version 5 has shown good psychometric properties [21, 22]. Participants indicate their level of agreement with the statements using a five-point Likert scale ranging from never = 1 to very often = 5. The score for each subscale ranges from 10 to 50. An overall score of 22 or less is indicative of low levels; a score of 23 to 41 is indicative of moderate levels; and a score of 42 or more is indicative of high levels of compassion satisfaction, secondary traumatic stress and burnout. Thus, the higher the score, the greater the compassion satisfaction, secondary traumatic stress and burnout.

#### Resilience CD-RISC-10

Is the shortened version of the original 25-item CD-RISC version. It is an unidimensional self-reported scale consisting of 10 items measuring resilience and having a high internal consistency (Cronbach’s alpha = 0.88). None of the items are reverse scored. Respondents rate items on a 5 point Likert scale, ranging from 0 (not true at all) to 4 (true nearly all the time). The overall score is calculated by summing all 10 items. A higher score indicates higher resilience [23].

#### Utrecht work engagement scale UWES-9

Is the subscale of the full version **UWES-17** which has shown robust psychometric properties [24]. It’s a brief valid questionnaire with 9-item scale developed to measure individual’s work engagement. Additionally, it consists of three dimensions: Absorption, Dedication and Vigor. Each item is scored on a six-point Likert scale ranging from 0 (never encountered situation) to 6 (situation encountered daily). An average score is obtained for each dimension and for the global scale. Thus, a higher score reveals higher level of work engagement.

#### Statistical analysis

Statistical analyses were conducted using the Statistical Package for the Social Sciences (SPSS) for Windows version 23. For the qualitative data, variables were described with frequencies and percentages means with standard deviations for the quantitative data. One-way ANOVA and Student’s t-test were used to compare professional quality of life among different demographic and exposure groups. All significant variables from the univariate analysis (p≤0.2) were incorporated in the linear multivariate model. Linearity of quantitative variables was checked by the scatterplot method. We set the statistical significance threshold p value at 0.05.

### Ethical considerations

#### Institutional review board statement

The study was approved by the medical ethics committee of Farhat Hached University Hospital. Approval number: CER:20–2022

#### Informed consent statement

Written informed consent was obtained from all subjects involved in the study. Participants were free to refuse participation. Anonymity and confidentiality were ensured.

## Results

### General characteristics of the study respondents

A total of 274 professionals were enrolled with a mean age of 32.87±8.35 years. Among them 66.4% were women (sex-ratio = 0.5). Almost half of the sample (45.6%) were married, 49.6% were doctors and 40.5% were nursing professionals. About half of them (49.8%) indicated that they were full vaccinated. Nearly two thirds of respondents 66.8% mentioned that they work in the Farhat Hached university hospital and less than two thirds 61.4% had a work experience less than 5 years. More than the half (54.7%) worked in intensive care units and nearly 13.5% mentioned a significant increase in their workload related to the management of Covid-19 patients. More details are summarized in Table 1.

**Table 1:** Participants’ demographics and work-related characteristics (N = 274).

Demographics and work-related characteristics	(Mean±SD)/ n (%)
Age (years)	32.87±8.35
**Gender**	
Male	92(33.6)
Female	182(66.4)
**Job categories**	
Medical personnel: Senior Doctors	27(9.9)
Medical personnel: Junior Doctors	136 (49.6)
Nursing personnel	111 (40.5)
**Marital status**	
Single	142(52.2)
Divorced	6(2.2)
Married	124(45.6)
**Work experience**	
<5 years	164(61.4)
5–20 years	83(31.1)
> = 20 years	20(7.5)
**Healthcare facilities**	
Farhat Hached	183(66.8)
Sahloul	91(33.2)
**Departments**	
Intensive care units	150(54.7)
Covid-19 units	124(45.3)
**Covid-19 vaccine**	
Yes, one dose received	52(19)
Yes, two doses received	136(49.8)
No	85(31.1)

### Professional quality of life, self-reported resilience and work engagement characteristics

The mean scores for compassion satisfaction, secondary traumatic stress and burnout were 35.09±7.08, 29.72±7.62 and28.54±5.44, respectively. Moreover, most of participants’ responses corresponded on the moderate level (23–41): 79.6% (n = 215) for both of compassion satisfaction and secondary traumatic stress, 86.9% (n = 232) for burnout. Approximately less than 5% of participants experienced high levels of secondary traumatic stress and burnout.

The self-reported resilience overall score ranged from 1 to 40 with a mean score of 25.34±6.21.

The work engagement score was 35.27±9.93 with extreme values ranging from 0 to 94. More details are summarized in Table 2.

**Table 2:** Professional quality of life, self-reported resilience and work engagement scores (N = 274).

	Mean± SD	n (%)
**Professional quality of life**		
**Compassion satisfaction**	35.09±7.08	
< = 22		10(3.7)
23–41		215(79.6)
> = 42		45(16.7)
**Secondary traumatic stress**	29.72±7.62	
< = 22		43(15.9)
23–41		215(79.6)
> = 42		12(4.4)
**Burnout**	28.54±5.44	
< = 22		34(12.7)
23–41		232(86.9)
> = 42		1(0.4)
**Self reported resilience**	25.34±6.21	
**Work engagement**	35.27±9.93	

### Correlations between professional quality of life, self-reported resilience and work engagement

Table 3 illustrates correlations between the three ProQoL components, resilience and work engagement. Thus, all three components of ProQoL were significantly correlated with self-reported resilience and with each other expect for compassion satisfaction and secondary traumatic stress. In fact, burnout had a negative correlation with compassion satisfaction (r = -0.486, p<0.01) and a positive correlation with Secondary traumatic stress (r = 0.382, p<0.01).

**Table 3:** Correlations between the professional quality of life, self-reported resilience and work engagement.

	Compassion satisfaction	Secondary traumatic stress	Burnout	Resilience	Work engagement
**Compassion satisfaction**	**_**				
**Secondary traumatic stress**	r = 0.017	_			
**Burnout**	r = - 0.486*	r = 0.382*	_		
**Resilience**	r = 0.420*	r = - 0.122*	r = - 0.265*	_	
**Work engagement**	r = 0.557*	r = -0.105	r = -0.376*	r = 0.467*	_

r: Pearson correlation

*p < 0.01

Regarding self-reported resilience, it was significantly correlated with all aspects of professional quality of life (p < 0.01). Thus, It had a positive correlation with compassion satisfaction (r = 0.420) but negative correlations with secondary traumatic stress (r = -0.122) and burnout (r = -0.265).

Additionally, it was found to have a positive correlation with work engagement (r = 0.467) which similarly in turn showed a significant positive correlation with compassion satisfaction (r = 0.557, p<0.01) and a significant negative correlation with burnout (r = -0.376, p<0.01). Otherwise, it showed no significant correlation with secondary traumatic stress.

### Univariate analyses of factors associated with professional quality of life

Table 4 shows the differences in mean scores for each of the ProQoL component based on socio-demographic and occupational variables. In fact, there were significant differences by professional category for all three components of the ProQoL: thus, junior doctors scored significantly higher on burnout, while they scored significantly lower on compassion satisfaction for which senior doctors earned the highest score (p = 10^−4^, 10^-3^respectively). On the other hand, nursing professional experienced significantly higher secondary traumatic stress than junior doctors (29.71±7.62 Vs 28.63±6.66; p = 0.04). Similarly, women experienced significantly higher secondary traumatic stress than men (30.98±7.92Vs 27.23±6.3; p = 10^−4^). In addition, compassion satisfaction and secondary traumatic stress have been found to score higher with married professionals (p = 0.05 and0.03 respectively). Regarding work experience, univariate analyses showed that those with less than 5 years of work experience reported significantly higher compassion satisfaction scores (p = 10^−4^)but paradoxically they earned the highest scores on burnout(p = 0.04). Interestingly, HCWs who reported compliance with protective measures were found to have significantly higher scores of compassion satisfaction (p<10^−3^)and significantly lower scores of burnout (p = 0.034). Additionally, HCWs who experienced feelings of fear of getting or transmitting the Covid-19 infection were significantly associated with higher scores of secondary traumatic stress (0.024).

**Table 4:** Univariate associations of demographics and work-related characteristics on compassion satisfaction, secondary traumatic stress and burnout.

	Compassion satisfaction M±SD	Burnout	Secondary traumatic stress
		M ± SD	
			M ± SD
**Gender**			
Male	34.87±6.82	28.54±5.02	27.23±6.31
Female	35.20±7.23	28.55±6.2	30.98±7.92
p	0.71	0.9	10^−4^*
**Marital status**			
Single	34.12±6.61	29.25±5.76	28.62±6.97
Married	36.23±7.3	27.88±5.01	31.06±8.25
Separated or widowed	34.50±10.4	26±4.6	29.33±6.86
p	0.05*	0.06*	0.03*
**Job categories**			
Medical personnel: Senior Doctors	37±5.36	26.29±4.80	29.59±7.33
Medical personnel: Junior Doctors	33.48±6.49	29.88±5.34	28.63±6.66
Nursing personnel	35.08±7.08	28.54±5.44	29.71±7.62
p	10^−3^*	10^−4^*	0.04*
**Departments**			
Intensive care units	34.54±6.39	28.92±5.17	29.50±6.71
Covid-19 units	35.75±7.81	28.09±5.73	29.97±8.61
p	0.1*	0.213*	0.6
**Work experience**			
< 5 years	33.75±8.87	29.24±5.59	29.14±7.86
5–20 years	36.84±5.66	27.61±5.29	30.78±7.39
>20 years	35.10±7.15	27.11±4.94	29.77±7.67
p	10^−4^*	0.04*	0.2*
**Healthcare institutions**			
FarhatHached	35.60±7.18	28.72±5.52	29.94±8.26
Sahloul	34.09±6.81	28.18±5.28	29.27±6.17
p	0.09*	0.49	0.44
**Workload**			
No difference or decrease	34.31±7.13	28.33±5.38	29.33±6.26
Slight increase	35.96±6.97	28.39±5.68	29.66±8.29
Significant increase	32.58±6.9	29.61±4.33	30.67±6.87
p	0.019*	0.449	0.683
**Compliance with protective measures**			
Yes	36.24±5.99	27.93±5.66	29.27±8.17
No	33.49±9.63	29.84±4.621	31.36±6.71
Neutral	32.8±6.09	29.55±5.24	29.30±6.42
p	0.003*	0.034*	0.177*
**Fear of infection**			
Yes	34.87±6.82	28.55±6.2	27.23±6.31
No	35.20±7.23	28.54±5.02	30.98±7.92
p	0.71	0.9	10^−4^*
**Fear of transmitting the infection**			
Yes	35.12±7.15	28.80±4.95	30.42±6.76
No	35.10±6.94	27.79±7.02	27.07±10.07
p	0.9	0.224*	0.024*

*P<0.05

### Regression analyses of demographics and work-related characteristics on compassion satisfaction, burnout and secondary traumatic stress

The results of the linear regression analyses are presented in Table 5. Statistically significant variables in Tables 3 and 4 were used as candidate variables. Thus, regression analysis revealed that self-reported resilience (β = 0.14, p = 10^−3^) and work engagement (β = 0.39, p = 10^−3^) were positively and significantly associated with compassion satisfaction, while burnout sub-score was a negative predictor factor (β = -0.32, p = 10^−3^). This model explained 45% of the variance (adjusted r^2^ = 0.45). In addition, a work experience of less than five years, compassion satisfaction and secondary traumatic sub-scores were found to be significantly associated with burnout (β = -0.1, p = 0.04; β = -0.54, p = 10^−3^; β = 0.35, p = 10^−3^ respectively) explaining 48% of the variance (adjusted r^2^ = 0.48).

**Table 5:** Regression analysis of demographics and work-related characteristics on compassion satisfaction, burnout and secondary traumatic stress.

	Compassion satisfaction r^2^ = 0.45			Burnout r^2^ = 0.48			Secondary traumatic stress r^2^ = 0.24		
	β	p-value	CI95%	β	p-value	CI95%	β	p-value	CI95%
**Gender**							0.20	10^−3^	[0.17;3.00]
**Marital status**							0.19	10^−3^	[1.14;4.19]
**Work experience**				-0.10	0.04	[-1.66;-0.01]			
**Fear of transmitting the infection**							0.11	0.03	[0.04;0.44]
**Compassion satisfaction**				-0.54	10^−3^	[-0.55;-0.37]			
**Burnout**	-0.32	10^−3^	[-0.55;- 0.29]				0.39	10^−3^	[0.36;0.70]
**Secondary traumatic stress**				0.35	10^−3^	[0.18;0.38]			
**Resilience**	0.14	10^−3^	[0.04;0.28]						
**Work engagement**	0.39	10^−3^	[0.20;0.42]						

Similarly, in the multiple linear regression model for STS, variables with significant weights were female gender(β = 0.20, p = 10^−3^), married status(β = 0.19, p = 10^−3^), fear of transmitting the infection (β = 0.11, p = 0.03) and burnout (β = 0.39, p = 10^−3^).

Nevertheless, the variance explained by this model (24%, adjusted r^2^ = 0.24) was lower than in the regression models for CF and BO.

## Discussion

The ongoing Covid-19 pandemic is expected to have a significant psychological impact on HCWs mainly manifested in anxiety disorders, burnout and secondary traumatic stress [5]. Indeed, CF can have severe repercussions for both HCWs and patient. It not only leads to decreased mental and physical health, but also to deterioration in the safety and quality of care delivered by professionals involved in the care of Covid-19 patients [11, 18].

The primary objective of this survey was to measure the levels of and compassion fatigue, conceptualized in burnout and secondary traumatic stress, and compassion satisfaction as well as their possible differences based on socio-demographic and work characteristics by resilience and work engagement scores among HCWs staff at two Tunisian university hospitals. It is clear that participants reported moderate levels of compassion satisfaction, burnout and secondary traumatic stress. Our survey not only reveals that HCWs tend to have an overall moderate levels of compassion satisfaction and compassion fatigue but also highlights the significant contribution, as predisposing factors, of self-reported resilience, work engagement and individual characteristics such as gender, marital status and the fear of transmitting the infection in explaining the prevalence of compassion fatigue and compassion satisfaction within the Tunisian HCWs during the Covid-19 pandemic. To the best of our knowledge, this study appears to be one of the first to deal with this topic in the Tunisian context. Therefore, the outcomes of this research may be a valuable reference for international audiences.

Overall, the study respondents reported moderate levels of compassion satisfaction, burnout and secondary traumatic stress. These findings join those of a similar Tunisian study performed during the second wave of the Covid-19 pandemic among 250 HCWs in public and private healthcare facilities, revealing that the sampled HCWs experimented moderate levels of compassion satisfaction and compassion fatigue [25]. Of note, 5% of participants in our study experienced high levels of compassion fatigue which was in concordance with a prior study conducted by Cho Lee Wong et al [16]. In fact, in some care departments, such as intensive care units and emergency departments, especially in times of health crises such as the one we are currently facing, HCWs may work in a fast-paced environment. Thus, due to the continuous exposure to emotional exhaustion, to potential risk of being infected, to the fear of transmitting the infection to their loved ones and to shortage of the resources, all of these further compromise their professional quality of life [26]. Our study found a considerably higher prevalence of burnout and secondary traumatic stress within our sample compared to that observed among HCWs in Italy or Hong Cong [16, 26]. This finding is probably due to the heavy workload to which Tunisian health professionals have been exposed, with a mismatch between available resources and the number of patients admitted. Another likely reason for this finding is that Tunisian HCWs were not used to deal with such an emerging epidemic as in Hong Kong, for example, where professionals already had experience in dealing with the SARS epidemic in 2003. Moreover, when comparing Tunisia to Italy and China, a resource gap between developed and developing countries could explain our results. Hence, the Covid-19 pandemic has only exposed the failures and shortcomings of the health system and has consequently increased the suffering of HCWs [27]. In this context, an immediate priority for Tunisian healthcare authorities is to implement extraordinary measures in order to alleviate the physical burden as well as the psychological frustration that HCWs suffer during this critical phase and it must involve all Public Health actors to improve the management of the covid-19 crisis in a multisectoral strategic approach.

However, a previous study carried out in China by Wu et al. revealed lower levels of CF among HCWs working on the front lines compared to those working in non Covid-19 units during the current pandemic [28]. According to the study authors, this result is related to the fact that these professionals were more trained in barriers measures and risk management and thus appear to have a better sense of virus control.

Our study showed that higher resilience scores were associated with less burnout and secondary traumatic stress. Similarly, both compassion satisfaction and work engagement were significantly and negatively correlated with BO. Our findings imply that self-reported resilience, compassion satisfaction and work engagement might be protective factors against physical and psychological exhaustion among Tunisian HCWs. In fact, resilience and work engagement were found to be predictor factors of compassion satisfaction in the linear regression analysis. Similarly, compassion satisfaction was found to be a determinant factor of BO. These findings confirm those of the one similar Tunisian study that used the same valid tools [25]. Indeed, some prior studies conducted in Spain and Saudi Arabia strongly supported this hypothesis [29, 30].

An interesting survey carried out in the Philippines by Labrague et al. that examines the mediating role of resilience in the association between CF and frontline nurses’ job outcomes in term of job satisfaction and care quality revealing that resilience plays a fully mediating role in the relationship between CF and quality of care and a partially mediating role in the relationship between CF and job satisfaction [19]. Thus, this study highlights the role of resilience in supporting the psychological well-being of HCWs and thus in ensuring healthcare quality and safety.

Our study identified that multiple factors may contribute to the development of CF and may influence the professional quality of life including personal characteristics HCWs such as gender, work experience and the fear of transmitting the infection. Indeed, the broader research literature has commonly focused on the ways in which the above factors affect the three aspects of professional quality of life during the Covid-19 pandemic. In fact, the prevalence of secondary traumatic stress was significantly higher among women. It has also been identified as an independent predictor of secondary traumatic stress. This is in concordance with previous studies suggesting that women experienced greater psychological vulnerability compared to men during this pandemic [25, 31]. In addition, a study conducted by Liu et al. that analyzed the difference of post traumatic secondary stress prevalence in gender during Covid-19 outbreak in China demonstrated that female healthcare professionals were more susceptible to have re-experiencing symptoms than men [32]. This finding is consistent with prior studies, showing that after a traumatic incident, acute psychological disorders featuring intrusive memories are more common in women than in men and that a female preponderance was noted in moderate to high STS category [33]. Similarly, a Tunisian study conducted by Sediri et al revealed the same finding consisting in the high occurrence of anxiety and depressive disorders as well as STS among Tunisian women during the lockdown [34]. These findings could be attributed to biological origins, such as differences in personality traits or to genetic and hormonal factors [34].

One of the findings of this study is that single professionals experienced lower level of STS than others who were engaged. This is consistent with earlier research conducted in Spain in which being married was found to be a predictor factor for the occurrence of CF [7]. However, marital status was not found to be a predictor factor of CF in a study carried out in Uganda [35]. A few possible explanations for these findings are that marital status as a variable refers to social support. However, in the work environment, perceived social support such as having a stable partner and a stable relationship may not be a factor influencing CF. HCWs look for support from their colleagues and supervisors [36].

Additionally, our study found that fear of transmitting the infection the infection, but not fear of contracting it, was found to be a determinant factor of STS. In fact, fear was not only related to the perception of an increased risk of infection by health professionals, especially since vaccination was still in its beginning in Tunisia and the conduct was strict isolation of patients with the use of full personal protective equipment, so that health professionals felt responsible not only for themselves and their patients, but also for their families. Further, the perception of being a potential source of contamination leads to an increased fear of being a vector of the virus for their loved ones. This overwhelming feeling of fear is the provider of STS disorder. Our results align with earlier researches reported in the literature [16, 37]. This finding underlines the need for organizationally focused interventions with structural changes, as well as strategies to improve the work environment [38].

Similar to the findings of the single Tunisian study addressing this topic during the Covid-19 pandemic, there were significant differences by professional category for all three components of the ProQoL [25]. Thus, junior doctors scored significantly higher on burnout, while they scored significantly lower on compassion satisfaction for which senior doctors earned the highest score. Indeed, poor work experience (less than 5 years) was found to be a predictor factor of burnout in the linear regression analysis. Indeed, this is in concordance with previous studies demonstrating that junior HCWs with poor work experience may have limited coping strategies. Moreover, the finding aligns with the common belief that CF declines with increasing work experience [39]. Our outcomes might be explained by the fact that the experienced personnel are used to deal with the challenging conditions of the Tunisian health system. Moreover, although senior HCWs appear to be more susceptible to Covid-19 infection, they seem to have more skills and better sense of control compared to junior HCWs.

By contrast, a large number of studies during the pre-pandemic time demonstrated that burnout was more significantly prevalent among the most experienced nursing providers than among those with fewer years of experience [40, 41]. Further studies on this topic are needed to better understand this paradox.

In view of the high prevalence of CF among HCWs in the front lines and the lack of national programs targeting HCWs’ mental health well-being, further work appears to be timely to identify appropriate CF prevention strategies in Tunisia, including educational programs, resilience promoting interventions, practice sessions and compassion or coping skills programs in order to improve HCWs compassion satisfaction and therefore their quality of life.

Our survey seems to be the first Tunisian study to deal with compassion fatigue and its predictor factors among frontline HCWs. Despite, the rigorous methodology based on reliable tools, these research outcomes must be cautiously interpreted given few limitations: First, the cross-sectional nature of the data provided no insight into cause-effect conclusions, further longitudinal studies on this topic are needed in the future. Second, the present study was conducted in only one Tunisian governorate which limits the representativeness of our results. Furthermore, data were self-reported and might be subject to social desirability bias. Finally, we used the French version of the ProQOL5 scale. This measurement instrument, although widely used to evaluate the quality of professional life among health professionals, has not been validated in French.

## Conclusion

A substantial proportion of Tunisian frontline HCWs reported moderate to high levels of compassion fatigue as well as compassion satisfaction. Nevertheless, more resilience promoting interventions and more coping skills programs must be implemented to fulfill their psychological well-being needs. Policymakers need effective strategies to improve mental health and cope with critical situations as well as to increase the productivity of hospital staff. Thus, further studies must address this crisis comprehensively and find ways to reduce its destructive effects.

## Supporting information

S1 Data (SAV)
